# A Letter

**Published:** 1874-04

**Authors:** A. W. Harlan

**Affiliations:** Chicago


### Miscellaneous.

### A LETTER.

CHICAGO, Feb. 16th.

TO THE EDITOR OF THE DENTAL REGISTER:

In the February No. of the REGISTER appears an article with the heading "A New Method of Pivoting," by B. Bannister, giving a description of the manipulations to successfully perform the operation, and he hopes that dentists will give "my method" a trial.

This is not a *new method;* it has been described in the last edition of Harris' "Principles and Practice," in the chapter on vulcanite, pages 710–11. Again, in Richardson's "Mechanical Dentistry." I do not know who the author of the method is, nor does it matter; but "give the D—— his due."

A. W. Harlan.

